# Microevolution of Virulence-Related Genes in *Helicobacter pylori* Familial Infection

**DOI:** 10.1371/journal.pone.0127197

**Published:** 2015-05-15

**Authors:** Yoshikazu Furuta, Mutsuko Konno, Takako Osaki, Hideo Yonezawa, Taichiro Ishige, Misaki Imai, Yuh Shiwa, Mari Shibata-Hatta, Yu Kanesaki, Hirofumi Yoshikawa, Shigeru Kamiya, Ichizo Kobayashi

**Affiliations:** 1 Department of Medical Genome Sciences, Graduate School of Frontier Sciences, University of Tokyo, Minato-ku, Tokyo, Japan; 2 Institute of Medical Science, University of Tokyo, Minato-ku, Tokyo, Japan; 3 Department of Pediatrics, Sapporo Kosei General Hospital, Sapporo-shi, Hokkaido, Japan; 4 Department of Infectious Diseases, Kyorin University School of Medicine, Mitaka-shi, Tokyo, Japan; 5 Genome Research Center, NODAI Research Institute, Tokyo University of Agriculture, Setagaya-ku, Tokyo, Japan; 6 Department of Bioscience, Tokyo University of Agriculture, Setagaya-ku, Tokyo, Japan; New England Biolabs, Inc., UNITED STATES

## Abstract

*Helicobacter pylori*, a bacterial pathogen that can infect human stomach causing gastritis, ulcers and cancer, is known to have a high degree of genome/epigenome diversity as the result of mutation and recombination. The bacteria often infect in childhood and persist for the life of the host. One of the reasons of the rapid evolution of *H*. *pylori* is that it changes its genome drastically for adaptation to a new host. To investigate microevolution and adaptation of the *H*. *pylori* genome, we undertook whole genome sequencing of the same or very similar sequence type in multi-locus sequence typing (MLST) with seven genes in members of the same family consisting of parents and children in Japan. Detection of nucleotide substitutions revealed likely transmission pathways involving children. Nonsynonymous (amino acid changing) mutations were found in virulence-related genes (*cag* genes, *vacA*, *hcpDX*, *tnfα*, *ggt*, *htrA* and the collagenase gene), outer membrane protein (OMP) genes and other cell surface-related protein genes, signal transduction genes and restriction-modification genes. We reconstructed various pathways by which *H*. *pylori* can adapt to a new human host, and our results raised the possibility that the mutational changes in virulence-related genes have a role in adaptation to a child host. Changes in restriction-modification genes might remodel the methylome and transcriptome to help adaptation. This study has provided insights into *H*. *pylori* transmission and virulence and has implications for basic research as well as clinical practice.

## Introduction

The pathogenic epsilon-proteobacterium *Helicobacter pylori* is a major cause of human gastric diseases [1]. The *H*. *pylori* genome sequence exhibits a high degree of diversity even between closely related strains because of the high rate of mutation and recombination [2–6]. The epigenome is also diverse owing to changes in methyltransferase genes through various mechanisms [7–12].

The significance of such extreme genome/epigenome diversity is not understood in detail, although the ability to adapt to the human host has been often suggested. Evidence for effects of DNA modification on gene transcription in bacteria has been limited primarily because of lack of techniques to sensitively and accurately detect methylated DNA bases and to accurately measure transcripts. The Single-Molecule Real-Time (SMRT) sequencing technology now allows methylome decoding at the single base resolution [13, 14]. On the other hand, RNA-seq method in the next-generation sequencers allows accurate and sensitive measurements of transcriptome changes [15]. As a result, there are now an increasing number of reports on relationship between DNA methylation by modification enzymes and transcriptome in bacteria. For example, Type II restriction-modification (RM) systems affect global gene expression in *Escherichia coli* [16] and in *H*. *pylori* [17]. Type III restriction-modification systems affect expression of various genes in *Neisseria*, *Haemophilus* and *H*. *pylori* [10, 18, 19]. Type I restriction-modification systems affect gene expression in *H*. *pylori* [7] and *Streptococcus pneumoniae* [20]. There is also evidence that Type II modification enzymes affect *H*. *pylori* physiology [21–23].

There have been many studies of human familial transmission in attempts to identify transmission pathways. Early studies used electrophoresis-based methods or multi-locus sequence typing (MLST) based on seven conserved genes (~3 kb in total) [24, 25]. Recently, more sensitive whole genome (~1.6 Mb) sequencing was used to analyze the process of familial infection [26–28].

In order to gain insight into *H*. *pylori* adaptive evolution in a new human host, especially in a child who was supposed to be newly infected, we used whole genome sequencing of very closely related *H*. *pylori* strains from the same family, which are difficult to distinguish by standard MLST analysis [29] and estimated the order of intrafamilial infection and then investigated which genes have experienced amino acid changes.

## Results

### Families infected with *H*. *pylori* of the same or very similar MLST sequence type

In all, 19 *H*. *pylori* strains were isolated from five families (one strain per person) visiting a pediatrician in a hospital in Hokkaido, Japan (**Table 1**). Each family member was infected with *H*. *pylori* strains of the same or very similar sequence type as judged by standard MLST analysis (**S1 Table**): the mother and the offspring in families K-1 and K-2, the parents and offspring in families K-3 and K-4, and the children in family K-5 [29]. The likely direction of familial transmission in families K-1 and K-2 was from mother to offspring. The allele type numbers of some of the seven MLST genes were different in families K-3 and K-4; this is based on a single nucleotide substitution, however, so we regard them as very similar sequences. The MLST results did not make it clear whether the father or the mother was the *H*. *pylori* donor to the offspring. In family K-5, transmission was likely from outside the family, which might be independent among children from the same reservoir or might have been followed by inter-child transmission.

**Table 1:** Strains and mapping results.

Family	Strain^a^	Relationship	Age	Mapped nt (> x5)	Mapped %	Variation #^b^
K-1	K16	Father	39	1456954	92.8	35242
	**K17**	Mother	38	1456823	92.8	34456
	**K15**	Child	10	1457425	92.8	34540
K-2	K34	Father	51	1459303	92.9	35271
	**K35**	Mother	46	1419326	90.4	37269
	**K36**	Child	17	1419365	90.4	37305
	**K37**	Child	14	1419197	90.4	37288
K-3	**K26**	Father	43	1459932	93.0	35041
	**K27**	Mother	28	1460893	93.0	34981
	**K28**	Child	6	1458283	92.9	34968
K-4	**K29**	Father	48	1379964	87.9	37440
	**K30**	Mother	44	1378891	87.8	37389
	**K32**	Child	14	1377440	87.7	37387
	**K33**	Child	13	1378456	87.8	37422
K-5	K21	Father	46	1462911	93.1	35064
	K22	Mother	47	1452079	92.5	34581
	**K23**	Child	17	1455829	92.7	33890
	**K24**	Child	15	1457747	92.8	33882
	**K25**	Child	5	1457010	92.8	33823

a Strains with same sequence type within a family are underlined.

b Number of variations compared with F30 genome.

All these strains were shotgun sequenced by an Illumina high-throughput sequencer and mapped onto the known genome sequence of *H*. *pylori* strain F30 (**Table 1**), which belongs to the hspEAsia population (as do most isolates in Japan) [4, 30]. About 90% of nucleotides were mapped with coverage of more than five reads, with detection of ~35,000 nucleotide substitutions per strain. A lower mapping rate in the strains found in family K-4 can be explained by the lack of a cag pathogenicity island in their genomes. Little difference was observed when other hspEAsia genomes (F16, F32 and F57) [4] were used as a reference genome for mapping (**S2 Table**), so we used the mapping on *H*. *pylori* strain F30 for further analysis.

### Estimation of transmission pathways from the number of nucleotide substitutions

**Fig 1:**
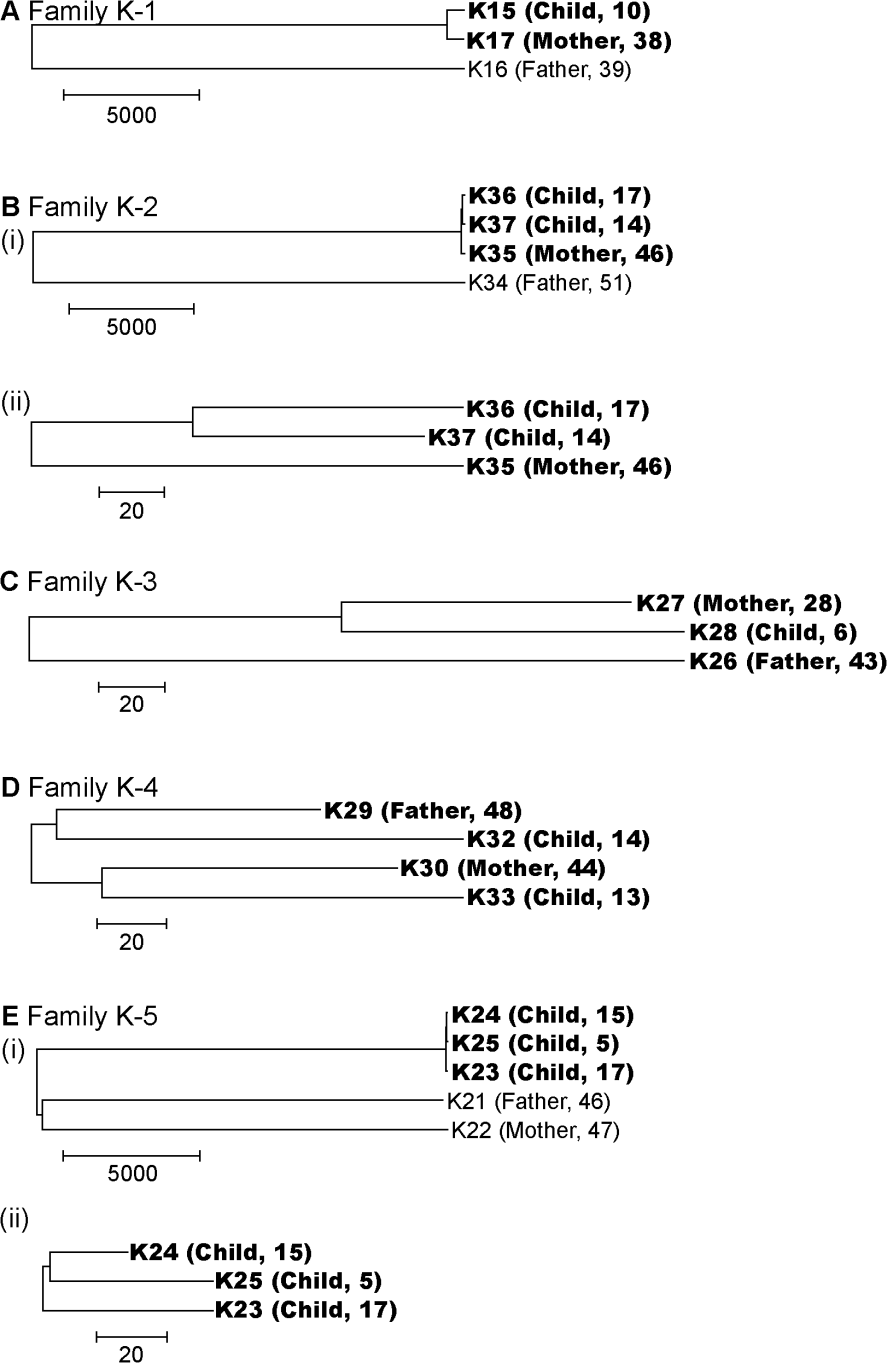
Phylogenetic trees of *H*. *pylori* genomes in the families. The number of nucleotide substitutions was used as the distance matrix. The relation and age of the host is added next to the strain name. (A) Family K-1. (B)(i) Family K-2. (ii) Strains with the same MLST sequence type in Family K-2. (C) Family K-3. (D) Family K-4. (E)(i) Family K-5. (ii) Strains with the same MLST sequence type in Family K-5. The bar indicates the number of nucleotide substitutions.

Nucleotide substitutions between each strain pair within a family were counted and used as the distance between compared strains (**S3 Table**) to construct a phylogenetic tree for each family (**Fig 1**). As expected, the number of nucleotide substitutions between strains of distantly related MLST sequence types was greater compared to the same or very similar MLST sequence type by one to three orders of magnitude. From the whole genome sequence information, various evolutionary relations were inferred between multiple strains of the same or very similar MLST sequence type within a family.

In family K-2, comparison of three strains with the same MLST sequence type showed the distance between the two strains from children K36 and K37 was, on average, 1.7-fold smaller compared to K35 (from mother) and K36 (from child) and to K35 (from mother) and K37 (from child) (**Fig 1B (ii)**). The difference was statistically significant (*P* < 10^–5^), supporting the hypothesis that the latest infection (transfer) occurred between the hosts of K36 and K37; i.e. transmission between children. (This inference is discussed in Discussion, below.)

A comparison of three strains with the same MLST sequence type in family K-3 showed the distance between K27 (from mother) and K28 (from child) is about half compared to K26 (from father) and K27 (from mother) and to K26 (from father) and K28 (from child) (**Fig 1C**). The difference is significantly greater compared to assuming a random distribution of substitution mutations (*P* < 10^–5^), suggesting the latest infection occurred between the hosts of K27 (mother) and K28 (child); i.e. transmission between mother and child. The likely direction is from mother to child, considering the general pathway of *H*. *pylori* infection. (See Discussion, below.)

No significant difference between distances was found for the four strains of very similar MLST sequence type in family K-4 (P > 0.01, **Fig 1D**).

There were three MLST sequence types corresponding to father, mother and offspring in family K-5 (see the preceding section). No significant difference between distances was detected for the three strains isolated from the offspring (P > 0.01, **Fig 1E (ii)**).

### Detection and classification of strain-specific nucleotide substitutions

All the nucleotide variations in strains with the same or very similar MLST sequence type were compared within each family for the detection of strain-specific nucleotide substitutions. Bases corresponding to the same site were compared, and assigned as a strain-specific substitution when only a single strain had a base different from the other strains in the comparison. In the case of family K-1, there were only two strains with the same sequence type, so we could not distinguish K15-specific from K17-specific substitutions and treated all the substitutions as strain-specific mutations.

We classified base substitutions to single nucleotide variations (SNVs) or clusters of nucleotide polymorphisms (CNPs) in order to estimate the frequency of recombination (**Table 2**). CNP is defined as a cluster of two or more substitutions separated by <200 bp and flanked by >200 bp of identical sequence on both sides and is considered as a sign of substitution by a recombination event [31]. Substitutions not clustered as CNPs were assigned as SNVs. A large number of CNPs was observed in family K-1, which accounted for about 85% of substitutions. The number of substitutions included in a CNP was, on average, about 11 for those of family K-1 but less than three in all the other strains.

**Table 2:** Strain specific nucleotide substitutions.

				Strain specific		Substitution	non-synonymous/	
Family	Strain	Relationship	age	nucleotide substitution	SNV/CNP	per CNP	synonymous/non-coding	Mutation rate^a^
K-1	K17 & K15^b^	Mother and Child	N/A^c^	1213	183/95	10.8	437/695/83	N/A
K-2	K35	Mother	46	182	154/12	2.3	96/62/24	N/A
	K36	Child	17	83	69/7	2.0	50/22/11	2.6 x 10^–6^
	K37	Child	14	71	54/7	2.4	40/20/11	2.5 x 10^–6^
K-3	K26	Father	43	291	214/27	2.9	186/64/41	N/A
	K27	Mother	28	87	74/6	2.2	46/24/17	N/A
	K28	Child	6	103	80/8	2.9	49/34/20	5.2 x 10^–6^
K-4	K29	Father	48	69	64/2	2.5	39/20/10	N/A
	K30	Mother	44	78	74/2	2.0	41/28/7	N/A
	K32	Child	14	110	89/8	2.6	77/20/33	4.0 x 10^–6^
	K33	Child	13	97	78/9	2.1	68/23/6	3.8 x 10^–6^
K-5	K23	Child	17	50	37/6	2.2	19/13/18	1.3 x 10^–6^
	K24	Child	15	17	17/0	N/A	12/4/1	0.7 x 10^–6^
	K25	Child	5	46	30/6	2.7	25/10/11	3.8 x 10^–6^

a Rate of SNV per site per year.

b K15 and K17 were counted together because we cannot assign strain specific substitutions by comparison of two strains.

c N/A, not applicable.

Strain-specific nucleotide substitutions were classified into nonsynonymous (changing amino acid), synonymous (retaining amino acid) and substitutions in noncoding regions. The mutation rate was calculated for isolates from children by dividing the number of SNVs (substitutions not included in CNPs) by their age in years, assuming *H*. *pylori* infection occurred soon after birth. The rate ranged from 0.7 x 10^–6^–5.2 x 10^–6^ per year per site (**S4 Table**). The value is comparable to those reported for earlier estimations for intra-family evolution [26, 31, 32].

### Genes with an amino acid substitution

We investigated which genes had experienced an amino acid substitution using function-based gene grouping with clusters of orthologous groups (COG) [33] (**Table 3, S5 Table**). The number of genes with at least one nonsynonymous substitution was counted. We found enrichment of amino acid substitutions in COG categories of M (cell wall/membrane/envelope biogenesis) and T (signal transduction) genes in multiple strains. The category M genes included those for outer membrane proteins (OMPs), lipopolysaccharide biosynthesis, lipoprotein-related proteins, penicillin-binding proteins in the cell wall and cell division proteins (**S6 Table**). Category T genes included *spoT* (included also in category K) and chemotaxis-related genes (included also in category N) (**S6 Table**). The genes with nonsynonymous substitutions included those decayed in hspEastAsia, including molybdenum-related genes (*mogA* and *modB*), and those decayed in hpEurope, including *ackA* for acetate formation [4]. The substitution mutations might represent a step in their decay.

**Table 3:** COG enrichment of genes with strain specific non-synonymous substitutions.

			K-1	K-2			K-3			K-4				K-5		
COG	Function	F30^a^	K15 & K17^b^	K35	K36	K37	K26	K27	K28	K29	K30	K32	K33	K23	K24	K25
C	Energy production and conversion	75	8	2	1	3	2	1	2	1	1	3	0	0	0	2
D	Cell cycle control and mitosis	20	0	2	1	1	1	0	0	1	0	0	1	0	0	0
E	Amino Acid metabolism and transport	97	9	3	2	0	7	4	2	3	6	1	2	0	0	0
F	Nucleotide metabolism and transport	39	3	1	2	1	1	0	0	1	0	2	0	0	1	0
G	Carbohydrate metabolism and transport	43	3	2	0	0	3	1	0	0	2	0	0	0	0	0
H	Coenzyme metabolis	77	5	2	2	1	5	1	0	3	1	0	0	0	1	2
I	Lipid metabolism	42	4	2	1	0	4	1	1	1	1	4	1	0	0	0
J	Tranlsation	122	7	6	1	2	7	3	2	2	0	6	5	1	0	0
K	Transcription	33	3	2	2	1	4	0	0	1	1	2	4	0	0	0
L	Replication and repair	88	9	7	1	2	4	4	3	1	2	6	2	1	1	0
M	Cell wall/membrane/envelop biogenesis	98	16	8	4	5	20*	3	3	4	8*	5	5	1	1	3
N	Cell motility	52	5	2	5	2	7	1	1	2	0	3	5	3	0	0
O	Post-translational modification	63	3	5	3	0	9	2	3	3	2	4	3	1	1	1
P	Inorganic ion transport and metabolism	62	10	4	1	2	6	2	1	0	4	1	3	1	1	0
Q	Secondary Structure	15	2	0	0	0	1	0	0	1	0	1	0	0	0	0
R	General Functional Prediction only	131	17	10	3	3	7	4	3	4	2	5	2	1	0	1
S	Function Unknown	72	7	1	1	0	8	4	2	0	0	5	4	0	0	2
T	Signal Transduction	25	5	2	5*	1	5	0	1	1	1	3	3	2	0	0
U	Intracellular trafficking and secretion	51	1	1	2	2	2	0	2	0	2	2	1	0	1	0
Z	Cytoskeleton	1	0	0	0	0	0	0	0	0	0	0	0	0	0	0
OMP	Outer membrane protein	46	18*	11*	3	4	14*	3	5*	3	4	8*	8*	3	2	6*
N/A		355	31	16	9	6	24	6	12	7	3	9	12	4	3	5
Sum		1607	148	78	44	36	107	40	38	39	32	62	53	18	12	16

a Strain used as reference strain for mapping.

b K15 and K17 were counted together because we cannot assign strain specific substitutions by comparison of two strains.

* Counts were tested by Fischer's exact test, P < 0.01

Genes not assigned to a COG category were also analyzed. Genes annotated as encoding OMPs [34] were the most frequently enriched with the amino acid changing mutations among the strains after transmission, as reported [27]. These included *alpAB*, *hofABDEF*, *horD*, *hopADFGINQZ*, *homBD*, *babAB* and other hypothetical OMPs (**Table 4 and S6 Table**).

**Table 4:** Select genes with an amino-acid substitution in *H*. *pylori* from children.

Group	Comment	Gene
Virulence-related	cagPAI (cag pathogenicity island)	*cagA*, *cagC*, *cagV*, *cagW*, *cagY*, *cag5*
	vacA homologs	*vacA*, vacA paralog, vacA paralog
	Helicobacter cysteine-rich protein	*hcpD*, *hcpX*
	Proteases	*htrA*, collagenase
	Type IV secretion system	*virB2*, *comH*
	Lewis antigen mimicry	*fucT*
	Others	*tnfα* (Tumor Necrosis Factor alpha-inducing protein),
		*ggt* (gamma-glutamyltranspeptidase)
Outer membrane protein	Hop family	*hopADFGINQZ*
	Hof family	*hofABDEF*
	Others	*alpAB*, *homBD*, *babAB*, *horD*
Restriction-modification	Type I	Restriction gene
	Type IIG	
	Type III	Restriction gene, Modification gene

Various known virulence factors were identified among them (**Table 4**). The *cagACTWY* genes reside on the cag pathogenicity island [35]; *vacA* is known to cause vacuolation in host cells [36] and *virB2* and *comH* are included in Type IV secretion systems, which are used for DNA import from the surrounding environment [37]. Cysteine-rich proteins [38] and HtrA protease [39] are involved in host interaction. The *tnfα* gene induces tumor necrosis factor alpha in the host [40] and gamma-glutamyltranspeptidase promotes pathogenesis [41–43]. The *fucT* gene is used for Lewis antigen mimicry and is important for immunity avoidance [44]. We also found amino acid substitutions in several restriction-modification genes (**Table 4**).

### Common and different patterns of evolution in familial transmission

We focused on three families involving children to gain insight into the steps of *H*. *pylori* adaptation to a human child host (**Table 5**and **S6 Table**). We emphasize that each lineage has a unique pattern of amino acid changing mutations but they all showed a change in *cag* and other virulence-related genes.

**Table 5:** Genes with non-synonymous mutations and annotations in *H*. *pylori* from children.

Family	Strain	Group/ COG category	Gene	Annotation	Locus tag of F30 ortholog
K-5	K23	Virulence-related	*cagA*	Cag pathogenicity island protein	HPF30_0779
		Virulence-related	*cagC*	Cag pathogenicity island protein	HPF30_0780
		Outer membrane protein	*hopQ*	Outer membrane protein	HPF30_0214
		Outer membrane protein	*hopI*	Outer membrane protein HopI	HPF30_0234
		Outer membrane protein	*alpB*	Outer membrane protein AlpB	HPF30_0426
		J	*sfhB*	Pseudouridine synthase	HPF30_0951
		M	*ftsI*	Cell division protein	HPF30_1449
		NT		Methyl-accepting chemotaxis protein	HPF30_0728
		NT	*mcpB*	Methyl-accepting chemotaxis protein	HPF30_1183
		NO	*flgA*	Flagellar basal body P-ring biosynthesis protein FlgA	HPF30_1345
		P	*fecA_2*	Iron dicitrate transport protein	HPF30_0524
		R	*rny*	Ribonuclease Y	HPF30_0573
			*hpaA*	Flagellar sheath adhesin HpaA	HPF30_0534
	K24	Virulence-related	*comH*	Periplasmic competence protein	HPF30_1399
		Outer membrane protein	*alpB*	Outer membrane protein AlpB	HPF30_0426
		Outer membrane protein	*hopN-2*	Outer membrane protein HopN2	HPF30_1068
		V	*hsdR_2*	Type I restriction enzyme R protein	HPF30_0485
		MU	*hefA*	Outer membrane protein HefA	HPF30_0721
		P		Catalase-related peroxidase	HPF30_0836
		H	*hemA*	Glutamyl-tRNA reductase	HPF30_1056
		F	*purB*	Adenylosuccinate lyase	HPF30_0276
	K25	Outer membrane protein	*babB*	Outer membrane protein	HPF30_0154
		Outer membrane protein	*hopL*	Outer membrane protein HopL	HPF30_0233
		Outer membrane protein	*alpB*	Outer membrane protein AlpB	HPF30_0426
		Outer membrane protein	*alpA*	Outer membrane protein AlpA	HPF30_0427
		Outer membrane protein	*babA*	Outer membrane protein BabA	HPF30_0441
		Outer membrane protein	*hopF*	Outer membrane protein HopF	HPF30_1043
		Virulence-related	*cagC*	Cag pathogenicity island protein	HPF30_0780
		O	*pcm*	Protein-beta-aspartate methyltransferase	HPF30_0297
		M	*cfa*	Cyclopropane fatty acid synthase	HPF30_0349
		C	*atpB*	F-ATPase subunit 6	HPF30_0502
		H	*mogA*	Molybdenum cofactor biosynthesis protein	HPF30_0532
		M		Putative outer membrane protein	HPF30_0673
		C	*ppa*	Pyrophosphate phospho-hydrolase	HPF30_0706
		H	*hemA*	Glutamyl-tRNA reductase	HPF30_1056
		M	*rfaJ-1*	Putative lipopolysaccharide biosynthesis protein	HPF30_1136
		R	*engB*	Probable GTP-binding protein EngB	HPF30_1459
			*hpaA*	Flagellar sheath adhesin HpaA	HPF30_0534
K-2	K36	Outer membrane protein	*hopQ*	Outer membrane protein	HPF30_0214
		Outer membrane protein	*alpA*	Outer membrane protein AlpA	HPF30_0427
		Outer membrane protein	*hopA*	Outer membrane protein HopA	HPF30_1066
		Virulence-related	*fucT*	Alpha-(1,3)-fucosyltransferase	HPF30_0677
		Virulence-related		Tumor necrosis factor alpha-inducing protein	HPF30_0731
		Virulence-related	*cagA*	Cag pathogenicity island protein	HPF30_0779
		Restriction-modification	*res-2*	Type III restriction enzyme R protein	HPF30_0033
		O	*groS*	Protein Cpn10	HPF30_0009
		H	*pnuC*	Nicotinamide mononucleotide transporter	HPF30_0110
		K	*cobB*	Regulatory protein SIR2 homolog	HPF30_0138
		V		ABC-transporter, ATP-binding domain	HPF30_0187
		C	*bisC-frg*	Biotin sulfoxide reductase BisC	HPF30_0341
		M	*capJ*	Type 1 capsular polysaccharide biosynthesis protein J	HPF30_0354
		R	*hypA*	Probable hydrogenase nickel incorporation protein HypA	HPF30_0464
		O	*tig*	PPIase	HPF30_0536
		TK	*spoT*	Penta-phosphate guanosine-3'-pyrophosphohydrolase	HPF30_0555
		FE	*prsA*	Phosphoribosyl pyrophosphate synthase	HPF30_0592
		NT		Methyl-accepting chemotaxis protein	HPF30_0728
		H	*bioF*	8-amino-7-oxononanoate synthase	HPF30_0729
		M	*pldA*	Phospholipase A1	HPF30_0822
		N	*fliG*	Flagellar motor switch protein G	HPF30_0946
		I	*dxr*	2-C-methyl-D-erythritol 4-phosphate synthase	HPF30_1079
		M	*rfaJ-2*	Putative lipopolysaccharide biosynthesis protein	HPF30_1087
		M	*rfaJ-1*	Putative lipopolysaccharide biosynthesis protein	HPF30_1136
		P		C(4)-dicarboxylates and tricarboxylates/succinate antiporter	HPF30_1152
		NT	*tlpA*	Methyl-accepting chemotaxis protein	HPF30_1179
		NT	*mcpB*	Methyl-accepting chemotaxis protein	HPF30_1183
		NT		Methyl-accepting chemotaxis transducer	HPF30_1218
		L	*topA*	DNA topoisomerase I	HPF30_1195
		U	*comB1*	ComB8 competence protein	HPF30_1259
		U	*comB6*	NADH-ubiquinone oxidoreductase subunit	HPF30_1260
		F		Putative endonuclease, split and separated by inversion, N-terminus part	HPF30_1272
		J	*rpsB*	30S ribosomal protein S2	HPF30_1429
	K37	Outer membrane protein	*hopQ*	Outer membrane protein	HPF30_0214
		Outer membrane protein	*hopL*	Outer membrane protein HopL	HPF30_0233
		Outer membrane protein	*hofG*	Putative outer membrane protein	HPF30_0425
		Outer membrane protein	*alpB*	Outer membrane protein AlpB	HPF30_0426
		Virulence-related	*cagA*	Cag pathogenicity island protein	HPF30_0779
		Restriction-modification	*res-2*	Type III restriction enzyme R protein	HPF30_0033
		Restriction-modification		Type IIG restriction-modification enzyme	HPF30_0661
		F	*pyrF*	Orotidine 5'-phosphate decarboxylase	HPF30_0005
		M	*tonB*	Siderophore-mediated iron transport protein	HPF30_0059
		J	*rpsK*	30S ribosomal protein S11	HPF30_0105
		K	*rpoB*	DNA-directed RNA polymerase subunit beta/beta	HPF30_0196
		P		Carbonic anhydrase	HPF30_0205
		M		Putative lipopolysaccharide biosynthesis protein	HPF30_0284
		C	*bisC-frg*	Biotin sulfoxide reductase BisC	HPF30_0341
		C	*ackA*	Acetokinase	HPF30_0435
		P		Iron(III) dicitrate transport protein	HPF30_0645
		L	*rnhA*	Ribonuclease H	HPF30_0667
		V	*hefC*	Cytoplasmic pump protein of the hefABC efflux system HefC	HPF30_0719
		MU	*hefA*	Outer membrane protein HefA	HPF30_0721
		M	*pbp-1a*	Penicillin-binding protein 1A	HPF30_0730
		NU	*flhA*	Flagellar biosynthesis protein FlhA	HPF30_0891
		J	*infB*	Translation initiation factor IF-2	HPF30_0898
		D	*minC*	Septum formation inhibitor	HPF30_0903
		L	*ruvB*	Holliday junction ATP-dependent DNA helicase RuvB	HPF30_0909
		R		Oligopeptide permease integral membrane protein	HPF30_1044
		NT	*mcpB*	Methyl-accepting chemotaxis protein	HPF30_1183
		C		Ferrodoxin-like protein	HPF30_1378
K-3	K28	Outer membrane protein	*hopQ*	Outer membrane protein	HPF30_0214
		Outer membrane protein	*alpB*	Outer membrane protein AlpB	HPF30_0426
		Outer membrane protein	*hofE*	Outer membrane protein HofE	HPF30_0548
		Outer membrane protein	*hofB*	Outer membrane protein HofB	HPF30_0932
		Outer membrane protein	*hopN-2*	Outer membrane protein HopN2	HPF30_1068
		Virulence-related	*cagV*	Cag pathogenicity island protein	HPF30_0794
		Virulence-related	*cagW*	Cag pathogenicity island protein	HPF30_0795
		Virulence-related	*cagY*	Cag pathogenicity island protein	HPF30_0797
		Virulence-related	*cag5*	Cag pathogenicity island protein	HPF30_0800
		Virulence-related	*vacA*	Vacuolating cytotoxin A	HPF30_0448
		Restriction-modification	*mod-4*	Putative type III restriction enzyme M protein	HPF30_1273
		Restriction-modification		Type I restriction enzyme R protein	HPF30_0858
		Restriction-modification	*hsdR_3*	Type I restriction enzyme R protein	HPF30_1407
		L	*dnaG*	DNA primase	HPF30_0010
		R		Lipid A phosphoethanolamine transferase	HPF30_0020
		O	*clpX*	ATP-dependent Clp protease ATP-binding subunit ClpX	HPF30_0030
		I		Isoprenyl transferase	HPF30_0175
		J	*valS*	Valyl-tRNA synthetase	HPF30_0237
		E	*nifS_2*	NifS-like protein	HPF30_0338
		S	*truD*	tRNA-uridine isomerase D	HPF30_0415
		C	*ackA*	Acetokinase	HPF30_0435
		S		Uncharacterized protein	HPF30_0472
		M	*waaE*	Bifunctional protein HldE	HPF30_0475
		M	*amiA*	N-acetylmuramoyl-L-alanine amidase	HPF30_0558
		R	*rny*	Ribonuclease Y	HPF30_0573
		L	*uvrA*	Excinuclease ABC subunit A	HPF30_0629
		M		Putative outer membrane protein	HPF30_0673
		P	*kefB*	Glutathione-regulated potassium-efflux system protein	HPF30_0851
		O	*htrA*	Protease DO	HPF30_0869
		J	*infB*	Translation initiation factor IF-2	HPF30_0898
		NT	*tlpA*	Methyl-accepting chemotaxis protein	HPF30_1179
		O	*clpA*	ATP-dependent C1p protease	HPF30_1264
				Response regulator	HPF30_0871

#### (i) Family K-2: K36 and K37

K36 had specific nonsynonymous nucleotide substitutions in five motility genes, five signal transduction genes, *cagA*, *tnfα*, *hcpD*, *fucT*, *rfaJ-1/2*, and one Type III R (restriction) gene. K37 had these types of mutations in *cagA*, *hcpD*, the RNA polymerase subunit gene (*rpoB*) and two restriction-modification genes (Type IIG and Type III R) as well as four OMP genes.

#### (ii) Family K-3: K28

Among the 49 genes with at least one nonsynonymous nucleotide substitution specific to the child strain, there are four *cag* genes (*cagV*, *cagW*, *cagY* and *cag5*), *vacA*, *htrA*, five OMP genes, three restriction-modification genes (two Type I R and one Type III M) and *clpX/clpP* protease genes (**Table 5**).

#### (iii) Family K-5: K23, K24 and K25

Among the three strains isolated from the three children in family K-5, K24 had the fewest (*n* = 12) genes with an amino acid change, among which are a virulence-related gene (*comH*), a restriction-modification gene (Type I R), *purB* for nucleotide metabolism, a peroxidase gene and a heme biosynthesis gene. K23 had nonsynonymous substitutions in *cagAC* genes, four genes related to motility and chemotaxis and two genes related to signal transduction. K25 had nonsynonymous mutations in *cagC*, three genes (*ppa*, *rfaJ-1* and *cfa*) related to lipids, *atpB* for membrane ATP synthase and *pcm* for protein repair.

## Discussion

*H*. *pylori* is known for sequence diversity between different strains, but strains from the same lineage can be difficult to distinguish by the standard MLST analysis using only seven genes. We undertook whole genome sequencing and distinguished the strain-specific nucleotide substitutions for isolates with the same or very similar MLST sequence type from the same family. On the basis of sequence difference, we revealed the likely pathway of evolution between these strains in some cases. Furthermore, after analyzing the nonsynonymous mutations, we suggested the strategy of *H*. *pylori* evolution during infection.

Following the construction of phylogenetic trees based on SNPs in the whole genome sequences. We inferred the direction of transfer between family members and other details (**Fig 1**). However, a recent study revealed a broad diversity in genome sequences in strains isolated from one specimen from one person [45]. The diversity represented in the above phylogenetic trees might well be accounted for by the diversity of lineages within an individual. In **Fig 1B(ii)**, for example, the mother might have transferred one lineage (the ancestor of K36) to one child and transferred another, well diversified strain (the ancestor of K37) to the other child. In order to clarify transmission pathways accurately, we need genome sequences of multiple strains from one host.

The mutation rate was calculated on the basis of strain-specific SNVs. Most of the value was included with the mutation rate range in earlier calculations from whole genome information [26, 31, 32] and only the K24 strain was a little below the range (**S4 Table**). This difference might reflect the origin of *H*. *pylori* strains. Earlier work using whole genome sequences was with strains from South Africa [26], whereas strains isolated in Japan were used in the present study. Another possibility is recombination between lineages [45]. By contrast, work using 78 genes for analysis gave a much lower value for the mutation rate, using strains derived from USA, UK, Colombia, the Netherlands and South Korea [32]. This result might be related to the large difference in sequence diversity among genes: indeed, different genes can evolve at very different rates [4, 5]. More analysis of whole genomes and individual genes in strains from various regions is required to fully understand the apparent variation of mutation rate.

Genes with an amino acid change might provide insight into the adaptation process. Many of these genes are related to the surface structure of the *H*. *pylori* cell, including OMP genes, lipoprotein-related genes and *fucT*, as found in other work on intrafamilial transmission and intrahost transmission [27, 31]. *H*. *pylori* can attach to human gastric epithelial cells through various kinds of adhesion factors, including BabA (HopS), BabB, SabA (HopP), SabB, AlpA and AlpB. Protein BabA is one of the major adhesion molecules associated with severe pathogenesis in *H*. *pylori* infection, although *babA* expression was reported to disappear by six months after infection of Mongolian gerbils with nucleotide changes introducing a stop codon of the gene [46]. AlpA and AlpB were shown to contribute to laminin binding of *H*. *pylori* and to induction of inflammatory changes of gastric mucosa [47]. HopQ might be important in initial colonization and long-term persistence of *H*. *pylori* in the stomach by modulating the adherence to gastric epithelial cells [48]. Two different alleles of *hopQ* were shown to be associated significantly with the positivity of other virulence genes, including *cagA* and *vacA* [49, 50].

An unexpected finding was the occurrence of amino acid substitutions in many virulence genes other than OMP genes and other surface-related genes. The genes of *vacA* and *cagA* are well known as important virulence-related genes of *H*. *pylori*. Mutations in these virulence genes were detected in *H*. *pylori* isolates of one or two family member(s) in each family but not, in general, in the isolates of all members of a family. One interesting possibility responsible for this observation is that these changes in virulence factors are related to adaptation to children in intrafamilial transmission. A related finding is that CagA is the most reactive antigen recognized by *H*. *pylori*-positive sera from children [51].

Amino acid changes in restriction-modification systems were detected in the three families involving children (see the last section in Results). A restriction-modification system consists of DNA methyltransferase, a modification enzyme, and a restriction endonuclease. DNA methyltransferase transfers a methyl group to a specific DNA sequence in the genome, which likely affects global gene expression among others. The restriction enzyme destroys DNA lacking such specific methylation resulting in genetic isolation. Recent work demonstrated the restriction-modification systems in *H*. *pylori* frequently change their presence/absence, sequence specificity and expression to remodel the methylome [7, 10]. The mutations mentioned above could be related to adaptation to a new host through such epigenetics-driven adaptive evolution [52]. Substitution of target recognition domains of restriction-modification systems underlying drastic changes in recognition sequence [11, 12], however, cannot be detected, in principle, by the present method based on mapping a genome sequence and SNP analysis.

Mutations were found in earlier work comparing whole genome sequences of closely related strains, especially in OMP genes, which is consistent with the results presented here [27, 28, 31, 53]. Comparison of whole genome sequences of *H*. *pylori* isolated from grandfather, son and grandson of a family in England found amino acid changes in OMP genes [28]. Substitution mutations in OMP genes were found in inter-spouse transmission in Australia [27]. A mutation burst was found during the acute phase of *H*. *pylori* infection leading to mutational changes in OMPs and *cag*-related genes in humans and primates [53]. These results are consistent with the present study and we additionally found mutations commonly observed in isolates from children in two categories; i.e. virulence factors other than *cag*-related and restriction-modification enzymes. This difference might be caused because of isolation in young children, compared to isolation from adults in other studies, but we cannot exclude the possibility it is caused by differences in environment.

We assumed that the individual human hosts have driven the bacterial mutations in the above genes. Our procedures involved culturing the bacteria *ex vivo* as in almost all the works on bacterial variations. Have the *ex vivo* steps, as opposed to *in vivo* steps, induced or selected the mutations we observed here? We think that most of the strain-specific nonsynonymous substitutions were generated *in vivo* for the following reasons. First, many of the genes with those mutations also show rapid sequence changes in phylogeny giving long branches in their phylogenetic tree [4, 5]. A simplest interpretation is the host adaptation through a nonsynonymous mutation is repeated for many generations to result in the rapid evolutionary rate. Second, comparable mutation rates per year were obtained in this and other studies based on strain culture *ex vivo* (**Table 2**). This indicates that the number of mutations is approximately proportional to the years within a human body. This cannot be expected only through mutagenesis and selection *in vivo*. Third, the strain-specific nonsynonymous mutations are unique to each of the strains. For example, K23, but no other strains, carries such mutations in multiple *cag* genes and several motility/chemotaxis-related genes among 14 genes with annotation (**Table 5**). Many strains carry those strain-specific nonsynonymous mutations in OMP genes, but the OMP repertoire is quite diverse among the strains. The difference is not likely a result of mutagenesis and/or selection during *ex vivo* culture, during which we used a medium of the same recipe, especially, the same batch of horse serum (see Materials and methods). From these considerations, it is, at present, natural to interpret that most of these strain-specific non-synonymous mutations were introduced during the long-term (years of) growth in individual human stomachs although we cannot exclude some contribution of *ex vivo* growth.

In conclusion, our whole genome decoding of *H*. *pylori* strains from family members including children suggested adaptation of these bacteria to a new human host through mutations in virulence-related genes and restriction-modification genes in addition to OMP genes.

## Materials and Methods

### Ethics statement

This study was undertaken with approval from the Ethics Committees of Kyorin University, Tokyo (No. 537) and Sapporo Kosei General Hospital (H24-104). Written informed consent was obtained from the patients (5 years old or older) and also from their parents when the patients are minor.

### Strains

In all, 19 *H*. *pylori* strains were obtained from five families during April 2011—December 2012 in Sapporo Kosei General Hospital, Sapporo, Hokkaido, Japan. A single colony was isolated and subcultured on Brucella medium supplemented with 1.5% (w/v) agar and 7% (v/v) horse serum (BHS medium) under microaerobic conditions. The same batch of horse serum was used for the culture to minimize possible variation between cultures. Typing of strains was done initially by seven-gene MLST for all five families [29].

### Genome sequencing and mapping

After incubation for 48 h under microaerobic conditions at 37°C on BHS medium, the culture of *H*. *pylori* (about 5×10^8^ colony-forming units) was collected. A Wizard Genomic DNA purification kit (Promega, Madison, WI, USA) was used according to the manufacturer’s instructions to isolate genomic DNA. A DNA library for genome sequencing was constructed by Nextera XT (Illumina, CA, USA) and sequenced by HiSeq2500 (Illumina, CA, USA).

About 1.4×10^6^ reads (~×200 coverage) with a length of 100 bp in the form of paired ends were selected from each read data (DRA accession no. 002504) and mapped against the genome sequence of *H*. *pylori* strain F30 (accession no. NC_017365) by BWA [54]. Nucleotide substitutions were detected by SAMtools software [55] without misalignment filtering to avoid pseudo-negative detection. Lists of nucleotide substitutions were compared by customized Perl scripts for calculation of the distance between strain pairs and for detection of strain-specific nucleotide substitutions. Nucleotide sites with coverage of more than five reads for all the members of a family with the same or very similar MLST sequence type were used for the detection of nucleotide substitution. Classification of nucleotide substitution to nonsynonymous, synonymous or substitutions at noncoding regions were done according to the gene annotation of *H*. *pylori* F30 [4]. For the calculation of strain-specific substitutions, the substitutions in strains K17 and K15 in family K-1 were counted together because the family has only two strains with the same MLST sequence type and it is not possible to assign substitutions to either of the strains.

The significance of differences between distances among strains with the same or very similar sequence type was analyzed by generating a matrix assuming the same probability of nucleotide substitution accumulation for all strain pairs. Matrices were constructed 1×10^6^ times and the rank list of standard deviation was compared with the standard deviation of distances in the real data for calculation of the *P* value.

### COG enrichment

COG of genes in the *H*. *pylori* F30 genome was annotated by rpsblast [56]. Genes with strain-specific nonsynonymous substitutions were counted and the significance of COG enrichment was tested by Fisher’s exact test. A gene was counted only once even if it had more than two strain-specific nonsynonymous substitutions.

## Supporting Information

S1 Table Sequence type. (XLSX)

S2 Table Mapping and nucleotide variation detection. (XLSX)

S3 Table Distance matrix. (XLSX)

S4 Table Comparison of mutation rate. (XLSX)

S5 Table COG of genes in *H*. *pylori* strain F30 whole genome. (XLSX)

S6 Table Strain-specific nonsynonymous nucleotide substitutions. (XLSX)
